# Frailty in randomised controlled trials for dementia or mild cognitive impairment measured via the frailty index: prevalence and prediction of serious adverse events and attrition

**DOI:** 10.1186/s13195-023-01260-3

**Published:** 2023-06-13

**Authors:** Heather Wightman, Terry J. Quinn, Frances S. Mair, Jim Lewsey, David A. McAllister, Peter Hanlon

**Affiliations:** 1 School of Health and Wellbeing, University of Glasgow, 1 Horselethill Road, Glasgow, G12 9LX UK; 2 School of Cardiovascular and Metabolic Health, University of Glasgow, Glasgow, UK

**Keywords:** Frailty, Dementia, Cognitive impairment, Randomised controlled trials

## Abstract

**Background:**

Frailty and dementia have a bidirectional relationship. However, frailty is rarely reported in clinical trials for dementia and mild cognitive impairment (MCI) which limits assessment of trial applicability. This study aimed to use a frailty index (FI)—a cumulative deficit model of frailty—to measure frailty using individual participant data (IPD) from clinical trials for MCI and dementia. Moreover, the study aimed to quantify the prevalence of frailty and its association with serious adverse events (SAEs) and trial attrition.

**Methods:**

We analysed IPD from dementia (*n* = 1) and MCI (*n* = 2) trials. An FI comprising physical deficits was created for each trial using baseline IPD. Poisson and logistic regression were used to examine associations with SAEs and attrition, respectively. Estimates were pooled in random effects meta-analysis. Analyses were repeated using an FI incorporating cognitive as well as physical deficits, and results compared.

**Results:**

Frailty could be estimated in all trial participants. The mean physical FI was 0.14 (SD 0.06) and 0.14 (SD 0.06) in the MCI trials and 0.24 (SD 0.08) in the dementia trial. Frailty prevalence (FI > 0.24) was 6.9%/7.6% in MCI trials and 48.6% in the dementia trial. After including cognitive deficits, the prevalence was similar in MCI (6.1% and 6.7%) but higher in dementia (75.4%). The 99th percentile of FI (0.31 and 0.30 in MCI, 0.44 in dementia) was lower than in most general population studies. Frailty was associated with SAEs: physical FI IRR = 1.60 [1.40, 1.82]; physical/cognitive FI IRR = 1.64 [1.42, 1.88]. In a meta-analysis of all three trials, the estimated association between frailty and trial attrition included the null (physical FI OR = 1.17 [0.92, 1.48]; physical/cognitive FI OR = 1.16 [0.92, 1.46]), although higher frailty index values were associated with attrition in the dementia trial.

**Conclusion:**

Measuring frailty from baseline IPD in dementia and MCI trials is feasible. Those living with more severe frailty may be under-represented. Frailty is associated with SAEs. Including only physical deficits may underestimate frailty in dementia. Frailty can and should be measured in future and existing trials for dementia and MCI, and efforts should be made to facilitate inclusion of people living with frailty.

**Supplementary Information:**

The online version contains supplementary material available at 10.1186/s13195-023-01260-3.

## Introduction

Frailty is an age-related state in which an individual’s physiological reserve is depleted across multiple systems, leading to an increased risk of decompensation in response to stressor events [1]. Frailty is associated with a higher risk of adverse events such as hospitalisation, falls, delirium, nursing home admission, and mortality [2]. The concept of frailty is particularly relevant in the context of cognitive impairment. Frailty is common among people living with dementia [3, 4]. Furthermore, frailty has been found to be associated with the development of dementia, including among people with or without cognitive impairment at baseline [5, 6]. Autopsy studies also suggest that frailty may moderate the relationship between neuropathological findings and the clinical expression of dementia [7]. The identification of frailty in people with mild cognitive impairment (MCI) or dementia may be useful to inform prognosis as well as identify individuals who may benefit from targeted support or intervention.

Despite its clinical implications, frailty is rarely measured or reported in randomised controlled trials (RCTs), [8–10] including those for MCI and dementia. RCTs offer the most reliable estimates of treatment efficacy and directly inform clinical guidelines. However, there are concerns that many RCTs are not representative of their target populations [9–13]. Quantifying frailty within RCTs would allow better estimation of the representativeness of RCTs and potentially facilitate the assessment of treatment efficacy by different degrees of frailty [14]. However, identifying frailty in RCTs is challenging for several reasons. First, there is no single universally accepted measure of frailty. Second, it is not clear whether the assessment of cognitive function should be intrinsic to the assessment of frailty or is a separate concept. Third, most RCTs do not include any explicit measure of frailty in their baseline assessment. Some frailty measures (such as the Groningen frailty indicator or the Kihon checklist) include an explicit assessment of cognition, whereas others (such as the frailty phenotype) include no cognitive variables [15, 16]. As a result, the prevalence and implications of frailty in RCTs for MCI and dementia are not clear.

One approach that may help to overcome the challenges of estimating frailty in RCTs is using the frailty index (FI) approach, originally described by Rockwood and Mitnitski [17]. An FI is a count of age-related deficits, the cumulative total of which defines an individual’s degree of frailty [18]. Importantly, there is no pre-specified list of deficits to be included in an FI and these can be selected based on the variables available within a given dataset. This flexibility inherent to the frailty index also allows for specific types of deficits (e.g. assessments of cognitive function) to be included or excluded from an FI. Several studies have retrospectively applied an FI to individual participant data from RCTs across a range of conditions [10, 19]. However, these studies have not assessed frailty in RCTs of MCI or dementia and have mostly included ‘physical’ deficits.

This study aims to construct an FI using baseline individual participant data (IPD) from RCTs for MCI or dementia. We will assess the prevalence of frailty and examine if frailty at baseline is associated with serious adverse events (SAEs) and trial attrition. We also aim to assess the impact of including cognitive function within the assessment of frailty by performing analyses using both a physical frailty index and an index which includes both physical and cognitive deficits.

## Methods

### Study design

This study quantified frailty using baseline IPD from three trials: two for MCI and one for dementia. For each trial, frailty was quantified using two frailty indices—one physical index (‘physical’ index) and one index which includes both physical and cognitive deficits (‘physical & cognitive’ index). The distribution of the FI (and the prevalence of frailty) and the relationship between frailty and serious adverse events (SAEs) and trial attrition were calculated. Analyses were conducted according to a pre-specified analysis plan (Additional file 1).

### Trial selection

Potentially eligible trials were identified first from clinicaltrials.gov as part of a wider project assessing comorbidity in trials across a range of index conditions. Eligibility criteria are described in detail elsewhere [20]. Briefly, eligible trials included 300 or more participants, had either no upper age limit or an upper limit > 60 years, and were phase 3 or 4 trials of pharmacological agents. We then narrowed these criteria to include trials for MCI or dementia. We included industry-sponsored trials for MCI or dementia for which IPD were available through the Yale University Open Data Access (YODA) repository. YODA provides access to trial IPD for third-party researchers.

Of the 2126 eligible registered trials, 30 were for dementia or MCI. Of these, three trials had IPD available with the YODA repository and were included in this analysis (a further 4 trials had IPD available from a different repository (Clinical Study Data Request); however, these trials had redacted the necessary data to construct the frailty index and so were excluded). The included trials were of galantamine. The original trials showed no difference in efficacy outcomes between treatment and control arms; therefore, treatment arm status was ignored in this secondary analysis. Two trials included community-dwelling participants with MCI (NCT00236431 and NCT00236574). The third trial included participants with severe Alzheimer’s disease (AD) dementia (NCT00216593).

The AD dementia trial ran from 2003 to 2008 and recruited people aged 40 or older with AD dementia rated as severe. People with dementia secondary to cerebrovascular disease were excluded. Full inclusion criteria can be found at https://clinicaltrials.gov/ct2/show/study/NCT00216593 and notably included ‘ability to be mobile (aided or unaided) with sufficient vision and hearing to comply with testing’. The two MCI trials were conducted between 2001 and 2003 and included people aged 50 or older. Inclusion and exclusion criteria can be found at https://clinicaltrials.gov/ct2/show/NCT00236431 and https://clinicaltrials.gov/ct2/show/NCT00236574, respectively. Of note, NCT00236431 excluded people with ‘clinically significant heart, lung, liver or kidney diseases’ and NCT00236574 excluded people with ‘Significant endocrine or metabolic disease’.

### Frailty index construction

We quantified frailty using an FI, based on Rockwood and Mitnitski’s ‘cumulative deficits’ model of frailty [17]. An FI is a count of age-related health deficits. There is no pre-specified list of deficits required to calculate an FI. Rather, deficits may be selected based on the available data providing they meet the following criteria: deficits must be health-related, must increase in prevalence with age, and must not saturate too early. Deficits must cover a range of physiological systems and indices must contain a minimum of 30–40 deficits [18].

We constructed two frailty indices, one based on physical deficits and another combining both physical and cognitive deficits, using the standard methodology described in Searle and colleagues’ ‘Standard Procedure for Creating a Frailty Index’ [18]. Deficits were identified from trial baseline assessments covering medical history, laboratory tests, neurological examination, and activities of daily living for the ‘physical FI’. The ‘physical & cognitive FI’ contained these same deficits plus additional deficits quantifying cognitive function.

Each individual deficit is defined in Additional file 1, with the data sources for each described below. Deficits were scored from 0 to 1. Where the deficit was binary, for example if a medical condition was present or absent, a score of 0 was given for the absence of a deficit and a score of 1 was given if the deficit was present. Where deficits were ordinal, scores were scaled between 0 and 1 (e.g. 0, 0.25, 0.5. 0.75, and 1 for a 5-level variable). Each FI was calculated as a ratio of the number of deficits an individual accumulated to the total number of deficits considered in the index. This results in a score between 0 and 1, with higher values indicating a greater degree of frailty. Where data were missing for a given deficit, this deficit was removed from both the numerator and the denominator (i.e. FI was calculated based on the number of available deficits for each individual). Where data were missing for > 20% of deficits, participants were excluded from the analysis.

#### Medical history

Details of medical history were redacted from the trial IPD. Therefore, we used concomitant medication data to identify medical conditions. These medication-based definitions were based on previous work quantifying multimorbidity in clinical trials and are described in detail elsewhere [20]. Eighteen conditions, meeting the FI criteria, were included in the FI.

#### Laboratory values

Laboratory deficits were based on baseline values for each trial.

#### Activities of daily living (ADLs)

Deficits related to activities of daily living were identified from baseline questionnaires used in each trial. The MCI trials shared the same assessment tool for ADLs—the Alzheimer’s Disease Cooperative Study-Activities of Daily Living Adapted to MCI (ADCS-ADL/MCI). The AD dementia trial recorded ADLs using the Minimum Data Set–Activities of Daily Living (MDS-ADL) questionnaire. Deficits were selected based on FI criteria and to cover a range of functional domains.

#### Physiological and clinical measurements

Deficits in blood pressure, body mass index, electrocardiographic abnormalities, and polypharmacy were also included. Polypharmacy was defined as taking five or more medications [21]. Relevant deficits were selected from baseline neurological examinations (e.g. muscle weakness, sensory impairment), which were recorded as ‘normal’ or ‘abnormal’.

#### Cognitive deficits

Cognitive deficits were selected from the Alzheimer’s Disease Assessment Scale–Cognitive Subscale (ADAS-Cog) for the AD dementia trial and the Mini-Mental State Examination (MMSE) for the MCI trials. We identified elements from each questionnaire assessing equivalent cognitive domains. A resulting five cognitive deficits—relating to the domains of orientation, memory, language, executive function, and constructional praxis—were included.

### Outcomes

#### Serious adverse events

SAEs are defined as any event occurring during the trial (regardless of cause or suspected relationship to the trial treatment) that results in death, serious risk to life, hospitalisation, congenital anomalies, or permanent disability [22]. For each trial participant, we assessed incident SAEs along with time to first event.

#### Trial attrition

Trial attrition was considered as withdrawal from the trial prior to its stipulated endpoint, for any reason [23].

### Statistical analysis

#### Frailty index distribution

Trial data were held within a secure repository which does not permit the export of individual-level data. Therefore, in order to report the prevalence and distribution of frailty, we summarised the FI distributions statistically. For each FI in each trial, we fitted lognormal, gamma, Weibull, and generalised gamma distributions. The fit of each distribution was compared using Kolmogorov–Smirnov tests. We found that the more flexible generalised gamma distributions fitted the data well for all trials. The parameters of this distribution were then exported from the YODA platform.

The cumulative density function of each distribution was used to summarise the overall distribution of the FI. To aid interpretation and comparison with previous literature, we also categorised frailty as ‘robust’ (FI < 0.12), ‘mild frailty’ (FI 0.12–0.24), ‘moderate frailty’ (FI 0.24–0.36), and ‘severe frailty’ (FI > 0.36). These cut-points were based upon the electronic frailty index in use within primary care systems within the UK [21]. We also calculated the 99th centile of each frailty index, as this has been previously used to indicate the upper limit of frailty within a population [24].

#### Association between frailty and SAEs

The association between frailty and the incidence of SAEs was assessed using Poisson regression. The model was adjusted for age and sex and included an offset for observation time. Participants were censored at first SAE or end of follow-up, whichever occurred first. The output of this model was the incidence rate ratio (IRR) of SAEs per 0.1-unit increase in FI.

For each FI, coefficients and their standard errors for each trial were pooled in a random-effects meta-analysis.

#### Association between frailty and trial attrition

The association between frailty and trial attrition was modelled using logistic regression. The model was adjusted for age and sex. The model output was the odds ratio (OR) for trial attrition per 0.1-unit increase in the frailty index. As for SAEs, estimates for each trial were combined using random-effects meta-analysis.

In post hoc analyses, we fitted interaction terms for sex and FI to models for SAEs and trial attrition to assess whether relationships between frailty and outcomes were different between men and women.

## Results

### Trial descriptive statistics and frailty indices

The three trials for which IPD were available through the YODA repository, two for MCI and one for dementia, are summarised in Table 1.

**Table 1:** Details of included trials

Trial	Trial ID	*N* participants	*N* excluded (> 20% missing data for frailty index)	Length of follow-up	Mean age (years)	% Female
**AD dementia trial**	NCT00216593	408	0	24 months	83.32	80.9
**MCI trial 1**	NCT00236431	987	0	12 months	69.50	53.7
**MCI trial 2**	NCT00236574	1064	0	12 months	70.63	57.0

**Fig. 1:**
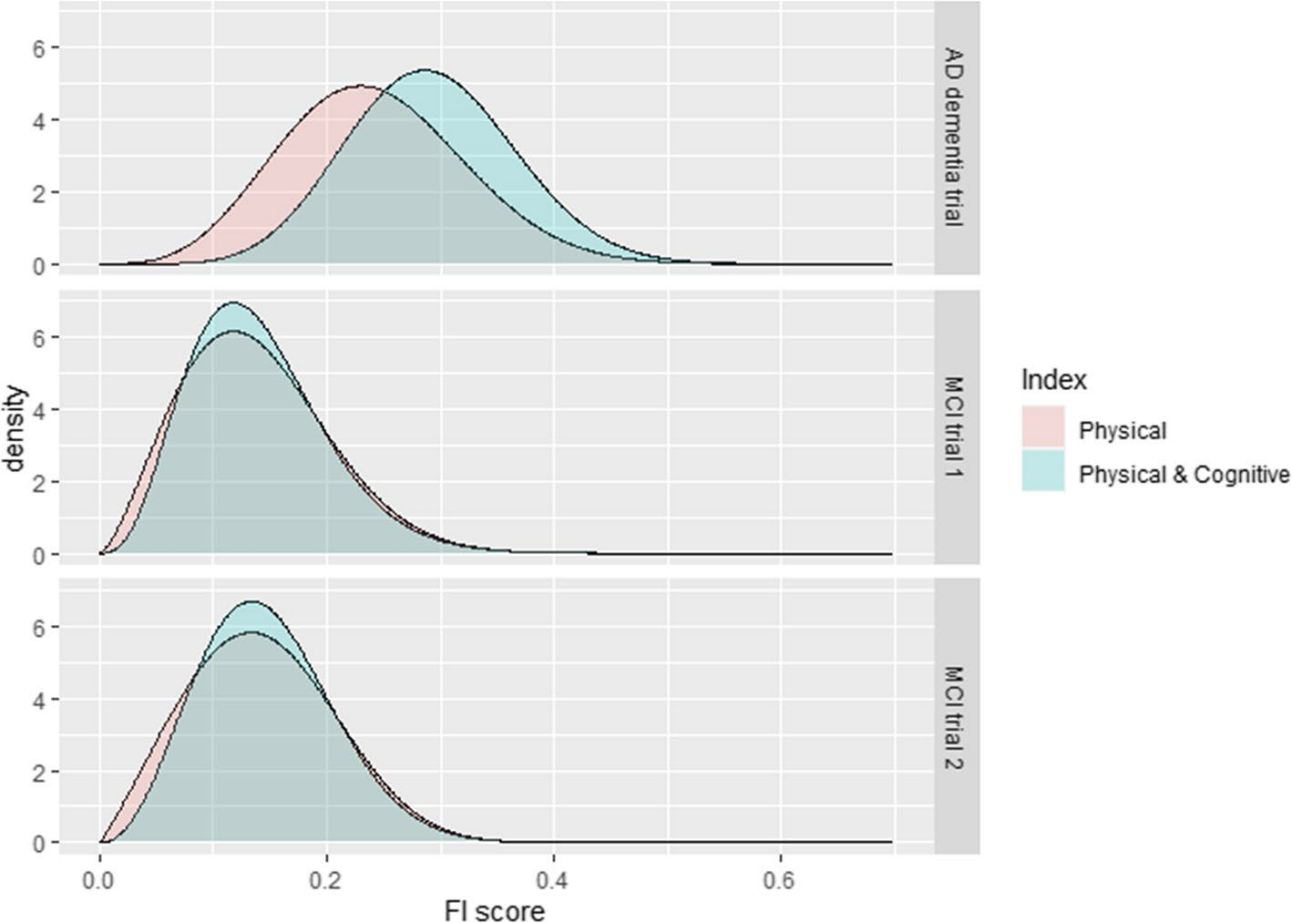
Distribution of the frailty index (comparing ‘physical’ and ‘physical & cognitive’ frailty indices): This figure shows the distribution of frailty in each of the included trials, as modelled using a generalised gamma distribution. Pink shading indicates the frailty index comprised only of physical deficits, and blue shading indicates the frailty index combining physical and cognitive deficits

The distributions of each frailty index in each trial were right skewed, as would be expected for a frailty index based on previous studies (Fig. 1). Mean frailty was higher in the AD dementia trial than in the MCI trials for both the physical index (mean FI 0.24 in AD dementia trial compared to 0.14 and 0.14 in the MCI trials, *p* < 0.0001 for both comparisons) and the physical & cognitive index (mean FI 0.29 in AD dementia trial compared to 0.14 and 0.15 in the MCI trials, *p* < 0.0001 for both comparisons), respectively (Table 2).

**Table 2:** Descriptive statistics of frailty indices

**Trial**	**Frailty index**	**Mean frailty index [SD]**	**99th percentile of frailty**	**Robust** (FI < 0.12)	**Mild** (FI 0.12–0.24)	**Moderate** (FI 0.24–0.36)	**Severe** (FI > 0.36)
AD dementia trial	Physical	0.24 (0.08)	0.44	5.5%	45.9%	41.1%	7.5%
	Physical & cognitive	0.29 (0.07)	0.47	0.5%	24.1%	57.5%	17.9%
MCI trial 1	Physical	0.14 (0.06)	0.31	43.2%	50.0%	6.7%	0.2%
	Physical & cognitive	0.14 (0.06)	0.30	41.6%	52.3%	5.9%	0.2%
MCI trial 2	Physical	0.14 (0.06)	0.30	38.7%	53.8%	7.5%	0.1%
	Physical & cognitive	0.15 (0.06)	0.30	35.0%	58.3%	6.6%	0.1%

In the AD dementia trial, the prevalence of frailty was greater when estimated using the ‘physical & cognitive’ frailty index, compared to the frailty index based on physical deficits alone (mean FI 0.29 and 0.24, respectively, *p* < 0.0001). In both MCI trials, the frailty prevalence was lower (5% moderate frailty and no severe frailty) than in the dementia trial. In the MCI trials, the prevalence of moderate frailty was similar between the two frailty indices (*p* = 0.56 and *p* = 0.18 for MCI trial 1 and MCI trial 2, respectively) (Table 2).

### Association between frailty and SAEs

**Fig. 2:**
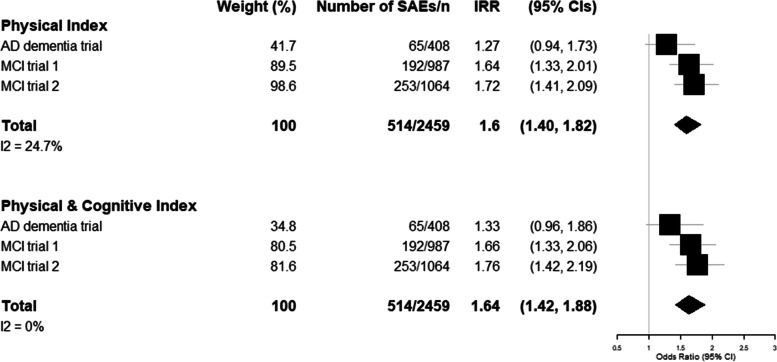
Association between frailty and incidence of serious adverse events. This figure shows the results of a random effects meta-analysis of the association between frailty index and incident serious adverse events. Incidence rate ratios (with 95% confidence intervals) show the results per 0.1-point increase in the frailty index

The association between frailty and SAEs is shown in Fig. 2. On meta-analysing the association across all three trials, there was an association between frailty and the incidence of SAEs for both indices (‘physical’ IRR 1.60 [95% CI 1.40–1.82] and ‘physical & cognitive’ IRR 1.64 [95% CI 1.42–1.88]).

### Association between frailty and trial attrition

**Fig. 3:**
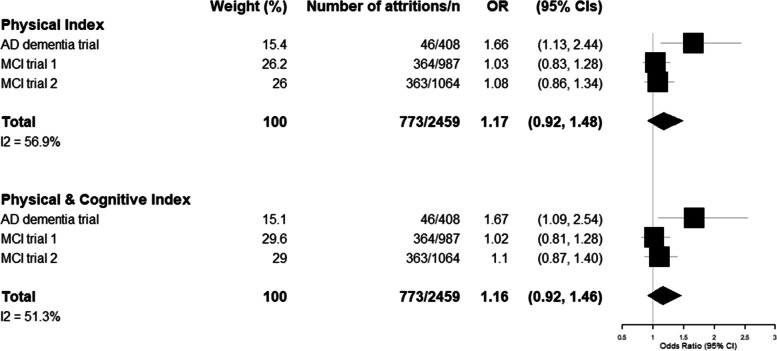
Association between frailty and odds of trial attrition. This figure shows the results of a random effects meta-analysis of the association between frailty index and trial attrition. Odds ratios (with 95% confidence intervals) show the results per 0.1-point increase in the frailty index

The association between frailty and trial attrition is shown in Fig. 3. A statistically significant association was found in the AD dementia trial (‘physical’ index OR 1.66 [95% CI 1.13, 2.44]; ‘physical & cognitive’ index OR 1.67 [95% CI 1.09–2.54]), but not in either of the MCI trials (MCI trial 1 ‘physical’ index OR 1.03 [95% CI 0.83–1.28]; ‘physical & cognitive’ index OR 1.02 [95% CI 0.81–1.28]) (MCI trial 2 ‘physical’ index OR 1.08 [95% CI 0.86–1.34]; ‘physical & cognitive’ index OR 1.10 [95% CI 0.87–1.40]). In a meta-analysis of all three, the association between frailty and trial attrition included the null (‘physical’ OR 1.17 [95% CI 0.92–1.48]; ‘physical & cognitive’ OR 1.16 [95% CI 0.92–1.46]). There was no significant interaction between sex and FI for either trial attrition or SAEs.

## Discussion

Using IPD from three trials for dementia or MCI, we found that it was feasible to measure frailty using a frailty index. Frailty was present to some degree across all three trials. However, severe frailty was absent in trials for MCI. Furthermore, the upper limit of frailty in all trials (estimated by the 99th percentile of the frailty distribution) was considerably lower than is seen in general population studies using the frailty index [24, 25]. Therefore, while frailty may be quantifiable and present in these trials, it is likely that severe frailty is under-represented. The inclusion of cognitive deficits within the frailty index resulted in a higher prevalence of frailty in the AD dementia trial, but little impact in the MCI trials. Frailty was associated with a higher incidence of SAEs. In a meta-analysis of all three trials, the estimated association between frailty and trial attrition included the null, although higher frailty index values were associated with attrition in the AD dementia trial, suggesting that frailty may impact attrition but only at more severe levels.

There were notable differences between the AD dementia trial and the MCI trials. Frailty prevalence was higher in the AD dementia trial. This may reflect a combination of higher frailty prevalence in people with dementia compared with MCI and differences in inclusion criteria for trials designed for dementia compared with MCI (for example, people living in nursing homes were specifically recruited in the dementia trial). Furthermore, higher FI values were associated with trial attrition in the AD dementia trial but not the MCI trial. This may reflect the relative lack of variability in the FI in the MCI trials (reducing the likelihood of detecting an effect), an association only between higher degrees of frailty and attrition, of a specific impact of frailty on attrition in the context of dementia.

The feasibility of measuring frailty in clinical trials using an FI and baseline IPD, demonstrated here for dementia and MCI, has been found in trials for other conditions, including hypertension, heart failure, chronic obstructive pulmonary disease (COPD), type 2 diabetes mellitus, and rheumatoid arthritis [10, 19, 26]. This suggests that reporting of frailty in trials should become more widespread, facilitating assessment of frailty prevalence and enhancing our understanding of the implications within trials.

Frailty is recognised to be common in people with dementia. One Canadian study examined the prevalence of frailty in nursing home residents with dementia (*n* = 42,828) using a 72-item frailty index and found that 69.6% of residents had an FI > 0.2 [27]. In the AD dementia trial, 75% of participants had an FI > 0.2. While not directly comparable, this suggests that, broadly, moderate frailty was well represented in the AD dementia trial. A study examining the prevalence of frailty in individuals with MCI (*n* = 3428) found that the mean FI was 0.14 (SD 0.08) [28]. This is comparable to the mean FI found in the MCI trials (0.14). However, the range of FI values in this study was 0.00–0.51. In this context, our finding that the 99th percentile of the FI was 0.3 in both MCI trials suggests that people with more severe frailty in the context of MCI may be excluded from the trials studied here.

The properties of the frailty index within the general population have been widely explored across a range of geographical locations and using different combinations of deficits within the frailty index. Properties such as the shape of the distribution and its upper limits have been consistent across different settings and applications [24, 29]. For example, assessment of frailty distributions in population samples from Canada, Australia, the USA, and Europe have shown a 99th centile of approximately 0.65 with little variation between geographical setting, age, and between institutionalised and non-institutionalised individuals [25]. In this context, the 99th centile in the AD dementia trial (0.44 or 0.47 depending on the exclusion or inclusion of cognitive deficits) is notably lower than might be expected.

The association found here between frailty and SAEs is consistent with the broad literature indicating frailty predicts a range of adverse health outcomes including mortality and hospital admission (which form the majority of SAEs in a trial setting) [2, 10]. Previous work examining frailty in trials for COPD, type 2 diabetes mellitus, and rheumatoid arthritis also found an association between frailty and the occurrence of SAEs [10]. To our knowledge, the relationship between frailty and trial attrition has not been widely studied. However, older age, poorer health, and higher levels of fatigue have been associated with higher rates of attrition [30–32]. In this context, it is perhaps surprising that the pooled relationship between frailty and trial attrition included the null. However, in the AD dementia trial, in which the trial population was older and both moderate and severe frailty were more common, frailty was associated with attrition. This could be suggestive of a non-linear relationship between frailty and the odds of trial attrition. Frailty may increase the probability of attrition but only above a certain threshold. While we explored non-linear terms in these data and found no such relationship, the absence of severe frailty in the MCI trials and the relative lack of variation within the FI in these trials means it may not have been possible to detect such an effect from these data alone.

The frailty index is one of a range of measures used to assess frailty [33]. The flexibility of the frailty index (in terms of what deficits are included) lends itself to secondary analysis (e.g. of trial data). This flexibility also allowed exploration of the impact of including or excluding cognitive measures in the assessment of frailty. There is an ongoing debate about whether cognitive impairment should be intrinsic to the definition of frailty or if ‘cognitive frailty’ should be considered as a distinct concept [34]. Our findings indicate that measuring frailty via an FI which excludes cognitive deficits will underestimate frailty in some people with dementia. This supports the principle that including a higher number of deficits in a frailty index will result in a more precise estimation of the degree of frailty [18]. Conversely, it could be argued that including cognitive deficits within a frailty index may overestimate frailty in the context of dementia (leading to high FI values even when other parameters are relatively spared). Despite this, the magnitude of association between frailty and both SAEs and trial attrition was similar regardless of the index used, suggesting that the utility of the frailty index for identifying people at higher risk of adverse outcomes is maintained with or without cognitive deficits.

Given the growing understanding of the importance of frailty in the development and progression of dementia, an assessment of frailty should be included in the design of clinical trials for dementia and MCI. This will allow for an improved understanding of the prevalence of frailty in clinical trials and for more powerful meta-analyses to be conducted to examine the association between frailty and trial outcomes. Additionally, a baseline frailty assessment may allow for the identification of those at a higher risk of attrition, allowing for measures such as home visits to be put in place to reduce potential attrition. This raises the question of what the optimal tool is to detect frailty in trials in general, and in dementia or MCI specifically. The frailty index, while flexible and applicable to existing data, relies heavily on comorbidities and additional insights may be gained by specifically collecting data on other frailty measures (e.g. physical measures such as the frailty phenotype or measures which explicitly include consideration of wider social vulnerability).

Improved reporting on the frailty of trial participants should be coupled with improved representation of people with moderate or severe frailty in trials, particularly for conditions such as dementia and MCI in which frailty is common and may influence treatment decisions. Under-representation leads to uncertainty as to the applicability of trial findings to people living with frailty. Improved representation of people living with frailty would better inform clinical guidelines as to the optimal treatments and balance of risks and benefits.

### Strengths and limitations

A strength of this study was that it used a well-recognised method for measuring frailty. Each index contained sufficient deficits, covering a wide range of physiological systems. However, as with many applications of the frailty index, the operationalisation relied on the available data, not on the prospective measurement of frailty. This meant that not all potentially important deficits such as a self-rating of health, visual acuity, deafness, and information on falls history were available to be included in the indices. Additionally, medical history had been redacted from the available data, and so the presence of comorbidities had to be inferred from concomitant medications. This has several implications. First, comorbidities had to be collapsed into broad groups treated with similar medications (e.g. cardiovascular disease was grouped and we did not attempt to differentiate between coronary heart disease, heart failure, etc.). Second, some conditions which are not primarily treated with specific medication (e.g. chronic kidney disease) were therefore not identifiable from medical history. Third, conditions for which medications are not always recommended (e.g. depression managed through non-pharmacological approaches) may be underestimated. Finally, in the context of dementia, some medications may have been considered inappropriate or de-prescribed which may lead to underestimation of some conditions. The frailty index is also just one of a wide range of frailty measures. Others, such as the frailty phenotype, could not be estimated from secondary analysis of trial data as the necessary variables are not measured. This is a disadvantage to this study due to the lack of consensus on which method is most suitable for quantifying frailty. Furthermore, the frailty index was limited to physical and cognitive variables and did not include related constructs such as social vulnerability, which may be associated with adverse events or trial attrition.

Another limitation of this study was in the clinical trials available. Only three trials were included in the analysis, and they were not a random sample (instead depending on which trials had been shared on the available platform). Therefore, while the findings of this study indicate that severe frailty may be under-represented in these trials, it is not clear to what extent these findings are generalisable to the wider body of trials for dementia or MCI.

## Conclusion

In summary, frailty can be measured in trials for dementia and MCI using a frailty index based on standard baseline trial measures and is associated with clinically meaningful outcomes. There is therefore potential for estimation and reporting of frailty to be incorporated into trial conduct as standard. This would allow widespread estimation of trial representativeness, assessment of the applicability of trial evidence to people living with frailty, and synthesis of trial findings to inform the treatment of people living with frailty.

## Supplementary Information

**Additional file 1.** Appendix.

## Data Availability

Trial data are available upon application to the Yale Open Data Access project and are held within a secure repository. Aggregate data approved for export from the repository are reported within the manuscript.

## Abbreviations

AD: Alzheimer’s disease
CI: Confidence interval
FI: Frailty index
IPD: Individual participant data
IRR: Incidence rate ratio
MCI: Mild cognitive impairment
OR: Odds ratio
