# Supplementary material for: Frailty in randomised controlled trials for dementia or mild cognitive impairment measured via the frailty index: prevalence and prediction of serious adverse events and attrition
Source: Alzheimers Res Ther. 2023 Jun 13;15:110. doi: 10.1186/s13195-023-01260-3 (PMC10262528; doi:10.1186/s13195-023-01260-3)
Supplement: Supplementary file 1 — Additional file 1. Appendix. [file 13195_2023_1260_MOESM1_ESM.docx]

**Appendix**

**‘Physical’ and ‘physical & cognitive’ frailty indices.**

|  | **AD Dementia Trial** | | **MCI Trials** | |
| --- | --- | --- | --- | --- |
|  | **‘Physical’ Index** | **‘Physical & Cognitive’ Index** | **‘Physical’ Index** | **‘Physical & Cognitive’ Index** |
| **Medical History** | **1. Acid related disorders**  Present = 1; Absent = 0 | **1. Acid related disorders**  Present = 1; Absent = 0 | **1. Acid related disorders**  Present = 1; Absent = 0 | **1. Acid related disorders**  Present = 1; Absent = 0 |
|  | **2. Arthritis**  Present = 1; Absent = 0 | **2. Arthritis**  Present = 1; Absent = 0 | **2. Arthritis**  Present = 1; Absent = 0 | **2. Arthritis**  Present = 1; Absent = 0 |
|  | **3. Anxiety**  Present = 1; Absent = 0 | **3. Anxiety**  Present = 1; Absent = 0 | **3. Anxiety**  Present = 1; Absent = 0 | **3. Anxiety**  Present = 1; Absent = 0 |
|  | **4. Cardiovascular disease**  Present = 1; Absent = 0 | **4. Cardiovascular disease**  Present = 1; Absent = 0 | **4. Cardiovascular disease**  Present = 1; Absent = 0 | **4. Cardiovascular disease**  Present = 1; Absent = 0 |
|  | **5. Diabetes**  Present = 1; Absent = 0 | **5. Diabetes**  Present = 1; Absent = 0 | **5. Diabetes**  Present = 1; Absent = 0 | **5. Diabetes**  Present = 1; Absent = 0 |
|  | **6. Dementia**  Present = 1; Absent = 0 | **6. Dementia**  Present = 1; Absent = 0 | **6. Dementia**  Present = 1; Absent = 0 | **6. Dementia**  Present = 1; Absent = 0 |
|  | **7. Epilepsy**  Present = 1; Absent = 0 | **7. Epilepsy**  Present = 1; Absent = 0 | **7. Epilepsy**  Present = 1; Absent = 0 | **7. Epilepsy**  Present = 1; Absent = 0 |
|  | **8. Glaucoma**  Present = 1; Absent = 0 | **8. Glaucoma**  Present = 1; Absent = 0 | **8. Glaucoma**  Present = 1; Absent = 0 | **8. Glaucoma**  Present = 1; Absent = 0 |
|  | **9. Gout**  Present = 1; Absent = 0 | **9. Gout**  Present = 1; Absent = 0 | **9. Gout**  Present = 1; Absent = 0 | **9. Gout**  Present = 1; Absent = 0 |
|  | **10. Inflammatory disorders**  Present = 1; Absent = 0 | **10. Inflammatory disorders**  Present = 1; Absent = 0 | **10. Inflammatory disorders**  Present = 1; Absent = 0 | **10. Inflammatory disorders**  Present = 1; Absent = 0 |
|  | **11. Osteoporosis**  Present = 1; Absent = 0 | **11. Osteoporosis**  Present = 1; Absent = 0 | **11. Osteoporosis**  Present = 1; Absent = 0 | **11. Osteoporosis**  Present = 1; Absent = 0 |
|  | **12. Pain**  Present = 1; Absent = 0 | **12. Pain**  Present = 1; Absent = 0 | **12. Pain**  Present = 1; Absent = 0 | **12. Pain**  Present = 1; Absent = 0 |
|  | **13. Parkinson's**  Present = 1; Absent = 0 | **13. Parkinson's**  Present = 1; Absent = 0 | **13. Parkinson's**  Present = 1; Absent = 0 | **13. Parkinson's**  Present = 1; Absent = 0 |
|  | **14. Asthma/COPD**  Present = 1; Absent = 0 | **14. Asthma/COPD**  Present = 1; Absent = 0 | **14. Asthma/COPD**  Present = 1; Absent = 0 | **14. Asthma/COPD**  Present = 1; Absent = 0 |
|  | **15. Thromboembolic disorders**  Present = 1; Absent = 0 | **15. Thromboembolic disorders**  Present = 1; Absent = 0 | **15. Thromboembolic disorders**  Present = 1; Absent = 0 | **15. Thromboembolic disorders**  Present = 1; Absent = 0 |
|  | **16. Thyroid disorders**  Present = 1; Absent = 0 | **16. Thyroid disorders**  Present = 1; Absent = 0 | **16. Thyroid disorders**  Present = 1; Absent = 0 | **16. Thyroid disorders**  Present = 1; Absent = 0 |
|  | **17. Schizophrenia**  Present = 1; Absent = 0 | **17. Schizophrenia**  Present = 1; Absent = 0 | **17. Schizophrenia**  Present = 1; Absent = 0 | **17. Schizophrenia**  Present = 1; Absent = 0 |
|  | **18. Urological disorders**  Present = 1; Absent = 0 | **18. Urological disorders**  Present = 1; Absent = 0 | **18. Urological disorders**  Present = 1; Absent = 0 | **18. Urological disorders**  Present = 1; Absent = 0 |
| **Laboratory Values** | **19. EGFR**  <30 = 1; <60 = 0.5; >60 = 0 | **19. EGFR**  <30 = 1; <60 = 0.5; >60 = 0 | **19. EGFR**  <30 = 1; <60 = 0.5; >60 = 0 | **19. EGFR**  <30 = 1; <60 = 0.5; >60 = 0 |
|  | **20. Haemoglobin**  <115 = 1 (men); <110 = 1 (women) | **20. Haemoglobin**  <115 = 1 (men); <110 = 1 (women) | **20. Haemoglobin**  <115 = 1 (men); <110 = 1 (women) | **20. Haemoglobin**  <115 = 1 (men); <110 = 1 (women) |
|  | **21. Platelet Count**  >450×10^9^/L *or* <150×10^9^/L = 1; 150–450×10^9^/L = 0 | **21. Platelet Count**  >450×10^9^/L *or* <150×10^9^/L = 1; 150–450×10^9^/L = 0 | **21. Platelet Count**  >450×10^9^/L *or* <150×10^9^/L = 1; 150–450×10^9^/L = 0 | **21. Platelet Count**  >450×10^9^/L *or* <150×10^9^/L = 1; 150–450×10^9^/L = 0 |
|  | **22. Liver Function**  AST \| >45u/L = 1; <=45u/L = 0    ALT \| >=50u/L (male), >=35u/L (female) = 1; <50u/L (male), <35u/L (female) = 0    (AST and/or ALT = 1) = 1; (neither AST nor ALT = 1) = 0 | **22. Liver Function**  AST \| >45u/L = 1; <=45u/L = 0    ALT \| >=50u/L (male), >=35u/L (female) = 1; <50u/L (male), <35u/L (female) = 0    (AST and/or ALT = 1) = 1; (neither AST nor ALT = 1) = 0 | **22. Liver Function**  AST \| >45u/L = 1; <=45u/L = 0    ALT \| >=50u/L (male), >=35u/L (female) = 1; <50u/L (male), <35u/L (female) = 0    (AST and/or ALT = 1) = 1; (neither AST nor ALT = 1) = 0 | **22. Liver Function**  AST \| >45u/L = 1; <=45u/L = 0    ALT \| >=50u/L (male), >=35u/L (female) = 1; <50u/L (male), <35u/L (female) = 0    (AST and/or ALT = 1) = 1; (neither AST nor ALT = 1) = 0 |
|  | **23. Sodium**  <133mmol/L = 1; >=133mmol/L = 0 | **23. Sodium**  <133mmol/L = 1; >=133mmol/L = 0 | **23. Sodium**  <133mmol/L = 1; >=133mmol/L = 0 | **23. Sodium**  <133mmol/L = 1; >=133mmol/L = 0 |
|  | **24. Calcium**  >=2.7mmol/L = 1; <2.7mmol/L = 0 | **24. Calcium**  >=2.7mmol/L = 1; <2.7mmol/L = 0 | **24. Calcium**  >=2.7mmol/L = 1; <2.7mmol/L = 0 | **24. Calcium**  >=2.7mmol/L = 1; <2.7mmol/L = 0 |
|  | **25. Cholesterol**  >=6.2mmol/L = 1; <6.2mmol/L = 0 | **25. Cholesterol**  >=6.2mmol/L = 1; <6.2mmol/L = 0 | **25. Cholesterol**  >=6.2mmol/L = 1; <6.2mmol/L = 0 | **25. Cholesterol**  >=6.2mmol/L = 1; <6.2mmol/L = 0 |
|  |  |  | **26. Glucose**  >11 mmol/L = 1, >7mmol/L = 0.5, <=7mmol/L = 0 | **26. Glucose**  >11 mmol/L = 1, >7mmol/L = 0.5, <=7mmol/L = 0 |
|  | **26. Ability to get dressed**  Ranked 0-1, where complete independence = 0 and total dependence or inability to perform task = 1 | **26. Ability to get dressed**  Ranked 0-1, where complete independence = 0 and total dependence or inability to perform task = 1 | **27. Ability to get dressed**  Ranked 0-1, where complete independence = 0 and total dependence or inability to perform task = 1 | **27. Ability to get dressed**  Ranked 0-1, where complete independence = 0 and total dependence or inability to perform task = 1 |
|  | **27. Ability to get around outside**  Ranked 0-1, where complete independence = 0 and total dependence or inability to perform task = 1 | **27. Ability to get around outside**  Ranked 0-1, where complete independence = 0 and total dependence or inability to perform task = 1 | **28. Ability to get around outside**  Ranked 0-1, where complete independence = 0 and total dependence or inability to perform task = 1 | **28. Ability to get around outside**  Ranked 0-1, where complete independence = 0 and total dependence or inability to perform task = 1 |
|  | **28. Eating**  Ranked 0-1, where complete independence = 0 and total dependence or inability to perform task = 1 | **28. Eating**  Ranked 0-1, where complete independence = 0 and total dependence or inability to perform task = 1 | **29. Prepare food**  Ranked 0-1, where complete independence = 0 and total dependence or inability to perform task = 1 | **29. Prepare food**  Ranked 0-1, where complete independence = 0 and total dependence or inability to perform task = 1 |
| **Activities of Daily Living (ADLs)** | **29. Bathing**  Ranked 0-1, where complete independence = 0 and total dependence or inability to perform task = 1 | **29. Bathing**  Ranked 0-1, where complete independence = 0 and total dependence or inability to perform task = 1 | **30. Balance a cheque book**  Ranked 0-1, where complete independence = 0 and total dependence or inability to perform task = 1 | **30. Balance a cheque book**  Ranked 0-1, where complete independence = 0 and total dependence or inability to perform task = 1 |
|  | **30. Personal Hygiene**  Ranked 0-1, where complete independence = 0 and total dependence or inability to perform task = 1 | **30. Personal Hygiene**  Ranked 0-1, where complete independence = 0 and total dependence or inability to perform task = 1 | **31. Clean common areas**  Ranked 0-1, where complete independence = 0 and total dependence or inability to perform task = 1 | **31. Clean common areas**  Ranked 0-1, where complete independence = 0 and total dependence or inability to perform task = 1 |
|  | **31. Ability to get around inside the home**  Ranked 0-1, where complete independence = 0 and total dependence or inability to perform task = 1 | **31. Ability to get around inside the home**  Ranked 0-1, where complete independence = 0 and total dependence or inability to perform task = 1 | **32. Clean laundry**  Ranked 0-1, where complete independence = 0 and total dependence or inability to perform task = 1 | **32. Clean laundry**  Ranked 0-1, where complete independence = 0 and total dependence or inability to perform task = 1 |
|  | **32. Using the toilet**  Ranked 0-1, where complete independence = 0 and total dependence or inability to perform task = 1 | **32. Using the toilet**  Ranked 0-1, where complete independence = 0 and total dependence or inability to perform task = 1 | **33. Use telephone**  Ranked 0-1, where complete independence = 0 and total dependence or inability to perform task = 1 | **33. Use telephone**  Ranked 0-1, where complete independence = 0 and total dependence or inability to perform task = 1 |
|  |  |  | **34. Keep appointments**  Ranked 0-1, where complete independence = 0 and total dependence or inability to perform task = 1 | **34. Keep appointments**  Ranked 0-1, where complete independence = 0 and total dependence or inability to perform task = 1 |
|  |  |  | **35. Go shopping**  Ranked 0-1, where complete independence = 0 and total dependence or inability to perform task = 1 | **35. Go shopping**  Ranked 0-1, where complete independence = 0 and total dependence or inability to perform task = 1 |
| **General** | **33. Blood pressure**  SBP >150mmHg = 1; SBP <= 150mmHg = 0 | **33. Blood pressure**  SBP >150mmHg = 1; SBP <= 150mmHg = 0 | **36. Blood pressure**  SBP >150mmHg = 1; SBP <= 150mmHg = 0 | **36. Blood pressure**  SBP >150mmHg = 1; SBP <= 150mmHg = 0 |
|  | **34. BMI**  <18.5kg/m^2^ *or* >30 kg/m^2^ = 1; >25kg/m^2^ = 0.5; 18.5-25 kg/m^2^ = 0 | **34. BMI**  <18.5kg/m^2^ *or* >30 kg/m^2^ = 1; >25kg/m^2^ = 0.5; 18.5-25 kg/m^2^ = 0 | **37. BMI**  <18.5kg/m^2^ *or* >30 kg/m^2^ = 1; >25kg/m^2^ = 0.5; 18.5-25 kg/m^2^ = 0 | **37. BMI**  <18.5kg/m^2^ *or* >30 kg/m^2^ = 1; >25kg/m^2^ = 0.5; 18.5-25 kg/m^2^ = 0 |
|  | **35. ECG**  Within normal limits = 0; Outwith normal limits = 1 | **35. ECG**  Within normal limits = 0; Outwith normal limits = 1 | **38. ECG**  Within normal limits = 0; Outwith normal limits = 1 | **38. ECG**  Within normal limits = 0; Outwith normal limits = 1 |
|  | **36. Polypharmacy**  >=5 medications = 1; <5 medications = 0 | **36. Polypharmacy**  >=5 medications = 1; <5 medications = 0 | **39. Polypharmacy**  >=5 medications = 1; <5 medications = 0 | **39. Polypharmacy**  >=5 medications = 1; <5 medications = 0 |
| **Neurological Examination** | **37. Gait**  Normal = 0; Abnormal = 1 | **37. Gait**  Normal = 0; Abnormal = 1 | **40. Gait**  Normal = 0; Abnormal = 1 | **40. Gait**  Normal = 0; Abnormal = 1 |
|  | **38. Coordination**  Normal = 0; Abnormal = 1 | **38. Coordination**  Normal = 0; Abnormal = 1 | **41. Coordination**  Normal = 0; Abnormal = 1 | **41. Coordination**  Normal = 0; Abnormal = 1 |
|  | **39. Sensory system**  Normal = 0; Abnormal = 1 | **39. Sensory system**  Normal = 0; Abnormal = 1 | **42. Sensory system**  Normal = 0; Abnormal = 1 | **42. Sensory system**  Normal = 0; Abnormal = 1 |
|  |  |  | **43. Muscle strength**  Normal = 0; Abnormal = 1 | **43. Muscle strength**  Normal = 0; Abnormal = 1 |
| **Cognitive Domains** |  | **40. Orientation**  Ranked 0-1; when 0 represents worst possible score and 1 represents best possible score |  | **44. Orientation**  Ranked 0-1; when 0 represents worst possible score and 1 represents best possible score |
|  |  | **41. Memory**  Ranked 0-1; when 0 represents worst possible score and 1 represents best possible score |  | **45. Memory**  Ranked 0-1; when 0 represents worst possible score and 1 represents best possible score |
|  |  | **42. Language**  Ranked 0-1; when 0 represents worst possible score and 1 represents best possible score |  | **46. Language**  Ranked 0-1; when 0 represents worst possible score and 1 represents best possible score |
|  |  | **43. Executive function**  Ranked 0-1; when 0 represents worst possible score and 1 represents best possible score |  | **47. Executive function**  Ranked 0-1; when 0 represents worst possible score and 1 represents best possible score |
|  |  | **44. Constructional praxis**  Ranked 0-1; when 0 represents worst possible score and 1 represents best possible score |  | **48. Constructional praxis**  Ranked 0-1; when 0 represents worst possible score and 1 represents best possible score |

**Analysis Plan**

# Protocol: Identifying frailty in clinical trials of drug interventions

October 2021

This analysis plan describes the proposed analysis of frailty, using the frailty index, in trials for dementia or mild cognitive impairment. It is an adaptation of the analysis plan from our previous publication assessing frailty in trials for type 2 diabetes, COPD, or rheumatoid arthritis.

Contents

[Protocol: Identifying frailty in clinical trials of drug interventions 1](#_Toc22887924)

[Background 1](#_Toc22887925)

[Exemplar conditions 2](#_Toc22887926)

[Trial eligibility criteria 2](#_Toc22887927)

[Trial summary data 2](#_Toc22887928)

[Frailty index: Identifying deficits 3](#_Toc22887929)

[Medical History Data 3](#_Toc22887930)

[Concomitant Medication 4](#_Toc22887931)

[Laboratory measurements 5](#_Toc22887932)

[Physiological measurements 5](#_Toc22887933)

[Functional limitations 5](#_Toc22887934)

[Symptoms 5](#_Toc22887935)

[Frailty index: Testing the appropriateness of deficits 5](#_Toc22887936)

[Calculating frailty index 6](#_Toc22887937)

[Comparing the impact of the availability of different measures 6](#_Toc22887938)

[Correlation between components of the frailty index 7](#_Toc22887939)

[Summarising frailty in trials 7](#_Toc22887940)

## Background

The term frailty describes an age-related decline in physiological reserve.^1^ People with frailty have a reduced capacity to return to homeostasis following a stressor event, putting them at increased risk of adverse health outcomes. Frailty carries an increased risk of mortality, hospitalisation, falls, and cognitive decline.^2^ It therefore has important implications for how we manage chronic disease.

Randomised controlled trials (RCTs) are the primary method through which the efficacy and safety of drug treatments for long-term conditions are tested. RCTs therefore directly influence clinical practice. It is not clear, however, to what extent people with frailty are represented in clinical trials. As such, the applicability of trial evidence to frail patients – and by extension the risks and benefits of treatments – is not clear.

This protocol outlines the methods for a project to demonstrate the feasibility of measuring frailty within individual participant data from RCTs, using a standardised frailty index approach.

There are a number of different approaches to identifying and quantifying frailty. One of the most widely used is the frailty index approach originally described by Rockwood and Mitnitski.^3^ The frailty index approach is particularly well suited to secondary analyses of existing data. A frailty index is a count of deficits (including morbidities, symptoms, blood abnormalities, functional limitations), which are associated with age and range across organ symptoms.^3^ A person’s frailty index is the arithmetic sum of their deficits divided by the total number of possible deficits (giving a value between 0 and 1). Moreover, a standardised procedure exists for constructing a frailty index from secondary analysis of existing data.^4^ It is therefore possible to apply the frailty index to data sources if a sufficient number of variables are available (typically 30-40) that fulfil pre-specified criteria for inclusion in a frailty index. Deficits can be selected from the available data provided: (i) they are biologically plausible (i.e. associated with health status); (ii) prevalence increases with age; and (iii) they do not saturate too early.^4^ The range of deficits included should also cover a range of organ systems.

## Exemplar conditions

Our previous publication, for which this analysis plan was originally developed, assessed three conditions.

- Type 2 diabetes mellitus (T2DM)
- Rheumatoid arthritis (RA)
- Chronic obstructive pulmonary disease (COPD)

This analysis has been published,^5^ and we now propose to extend this to trials for dementia or mild cognitive impairment. The approach used, and adaptations to the approach to apply to dementia or mild cognitive impairment, are described below.

## Trial eligibility criteria

We will analyse individual-level participant data (IPD) from industry-sponsored trials from two repositories: the Clinical Study Data Request (CSDR) and the Yale University Open Data Access (YODA) project. This analysis will consider trials pertaining to dementia or mild cognitive impairment. These trials are a subset of trials identified as part of a broader project assessing multimorbidity within trial IPD.^6^

This broader selection of trials were selected according to a pre-specified protocol (Prospero CRD42018048202). Briefly, eligible trials were registered with the US Clinical Trials register (clincialtrials.gov), had a start date on or after 1^st^ January 1990, were phase-2/3,-3 or 4, recruited ≥300 participants, had an upper age limit ≥60 years (or no maximum), and evaluated drugs for a selected set of chronic conditions.

Only randomised participants will be included in analyses.

## Trial summary data

For each trial included, we will summarise the following data:

- Drug and comparison
- Indication
- Sponsor
- Inclusion criteria
- Exclusion criteria
- Age/sex distribution of participants
- Ethnicity
- Socioeconomic status
- Number and type of comorbidities
- Start date
- Study location/site

## Frailty index: Identifying deficits

To identify deficits from clinical trial data, we will consider the following:

- Medical history data (i.e. comorbidities)
- Concomitant medication data (either to confirm/refine diagnoses from medical history, or to identify the presence of diagnoses in the absence of medical history data)
- Laboratory measurements
- Physiological measures (e.g. spirometry, walking speed)
- Functional limitations (identified using quality of life questionnaires)
- Symptoms (e.g. pain), taken from generic or disease specific questionnaires

### Medical History Data

Clinical trials often code medical history according to the MedDRA coding system. MedDRA is a highly specific standardised medical coding system which has been designed to facilitate sharing of regulatory information for medical products. There are a large number of MedDRA terms, and these are organised within a hierarchical structure. Some (e.g. chronic kidney disease, hypertension etc.) can be identified using existing groups of terms known as ‘Standardised MedDRA queries’ (SMQs). SMQs are described by MedDRA as “validated, pre-determined sets of MedDRA terms grouped together after extensive review, testing, analysis, and expert discussion”.^7^

Where baseline medical history is coded using MedDRA, we will apply pre-specified MedDRA terms to identify comorbidities. Where an SMQ exists to describe a condition, we will use this group of terms as described by MedDRA. Where no SMQ has been validated for a condition, we will manually select and pre-specify terms used to identify the condition.

The following comorbidities will be assessed as deficits. For conditions indicated with an * we will use Structured MedDRA queries to identify the deficit:

Musculoskeletal

- Gout
- Osteoarthitis
- Rheumatoid arthritis/other connective tissue disease
- Osteoporosis/osteopenia*

Renal/urological

- Chronic kidney disease*
- Urinary incontinence

Neoplastic

- Malignancy (any)*

Psychological

- Depression*

Cardiovascular

- Dyslipidaemia*
- Ischaemic heart disease*
- Hypertension*
- Cardiac arrhythmia
- Heart failure*
- Valve disorder
- Deep vein thrombosis/pulmonary embolism

Respiratory

- Chronic obstructive pulmonary disease

Metabolic/endocrine

- Thyroid disorders*
- Diabetes mellitus
- Anaemia

Hepatobiliary/gastrointestinal

- Hepatic disorders*
- Diverticular disease

Neurological

- Peripheral neuropathy*
- Stroke
- Epilepsy
- Parkinsonism
- Dementia

Ophthalmological

- Glaucoma

If a comorbidity appears to be missing in a trial, we will search the trial documentation to see if this type of condition was not asked about. Where this appears to be the case, the condition will not be included in the frailty index.

Where data on medical history is missing or redacted, we will use concomitant medication to identify broad groups of comorbidities from medication use. These ‘medication-based’ definitions will be based on pre-specified groups of medications, coded according to the World Health Organisation Anatomic Therapeutic Classification (WHO-ATC). These definitions were developed as part of a broader project assessing multimorbidity within clinical trials. The selection of these definitions is described in detail elsewhere.^6^

### Concomitant Medication

Where trials report medical history data, we will use concomitant medication to refine or expand upon these definitions, or include additional deficits. Examples may include:

- Polypharmacy (taking ≥5 medications, consistent with the cut-off used in the electronic frailty index by Clegg et al.)^8^
- Conditions or symptoms that may not be reported in medical history but may be inferred from medication use (e.g. pain, dyspepsia)

### Laboratory measurements

We will assess the following deficits:

- Anaemia (Hb below sex-specific reference range)
- Renal impairment (based on estimated Glomerular Filtration Rate (Modification of Diet in Renal Disease (MDRD) equation) where available)
- Hypoalbuminaemia (serum albumin below reference range)
- Systemic inflammation (elevated C-reactive protein or erythrocyte sedimentation rate)

Liver impairment (Fibrosis-4 index)

### Functional limitations

The primary source of data pertaining to function/activities of daily living is anticipated to be quality of life questionnaires. Specific questions will be selected from available questionnaires and used to identify deficits for inclusion in a frailty index. Examples from deficits from two commonly used questionnaires are shown below:

EQ-5D

- Mobility (‘I have severe problems walking about’ or ‘I am unable to walk about’)
- Self-care (‘I have severe problems washing or dressing myself’ or ‘I am unable to wash or dress myself’)

SF-36

- Climbing one flight of stairs (‘limited a lot’ or ‘limited a little’)
- Physical health problems limit the kind or work or activities you do (yes/no)

For trials of dementia of mild cognitive impairment, we andicipate that trials may use questionnaires or scales specific to these conditions. In this case, deficits related to activities of daily living or broader function will be selected from the available questions.

Responses will either be dichotomised (deficit present/absent) or graded in severity depending on the nature of the question.

### Symptoms

Symptoms will be identified from questionnaires/disease severity scores where available. We will use validated scores (e.g. MRC dyspnoea scale) to grade the severity of the deficit where available. Otherwise, symptoms will be coded as presence/absence.

### Cognitive deficits

Cognitive deficits will be selected from the cognitive assessment questionnaires used in each trial. Where trials use different questionnaires/assessment tools, we will attempt to harmonise these by selecting questions/assessments which test similar domains between questionnaires.

## Frailty index: Testing the appropriateness of deficits

To be included in a frailty index, deficits must fulfil three basic criteria:

- Biologically plausible
- Increase in prevalence with age
- Do not ‘saturate’ too early

To fulfil the first criteria, deficits will be based on comorbidities, laboratory deficits, and functional impairments. The choice will be informed by clinical judgement of the project team as well as reference to prior literature using the frailty index approach.

Our judgement of the second and third criteria will be based on established current literature on frailty. We will include deficits such as comorbidities which have been demonstrated to increase in prevalence with age and have been previously used as part of frailty indices. Similarly with functional deficits, these will be taken from validated quality-of-life and symptom questionnaires and previous literature will be used to confirm the relationship of deficits with age. This approach will be taken over a data-driven approach, as the prevalence of deficits in a trial context (subject to inclusion and exclusion criteria) may not reflect the true prevalence of a deficit, and its relationship with age, in the general population.

## Calculating frailty index

The frailty index will be calculated using previously described methods.^4^ Deficits will be scored 0 or 1 for binary deficits, or for ordinal variables (e.g. degree of activity limitation from EQ5D) values will be assigned based on severity (e.g. 1 (most severe), 0.5, 0 (absent)). For each trial participant, the total score for each deficit will be summed, and then divided by the total number of possible deficits. This will give an overall value between 0 and 1.

We will then produce summary statistics for the distribution of the frailty index for each trial, and across trials for each condition.

To assess the impact of missing data within the context of RCT data, we will calculate the frailty index first using complete data only, then by multiple imputation. Only deficits with <10% missing data will be imputed.

## Correlation between components of the frailty index

The overall frailty index is an unweighted count of all included deficits. In addition to this, we will divide deficits into comorbidities, functional measures (i.e. activities of daily living from quality of life scores) and physiological measures (e.g. laboratory measures, spirometry etc.). We will then assess the correlation between counts of each type of deficit (i.e. the correlation between number of comorbidities and number of functional deficits). We hypothesise that there will be a positive correlation (i.e. people with more comorbidities are more likely to have more functional and physiological deficits) but that correlation will not be perfect (i.e. comorbidity alone does not perfectly predict the degree of functional of physiological deficit). This will allow us to assess the potential impact of assessing a wider degree of functional and physiological measures in a trial setting.

We will also assess the correlation between the frailty index and:

- Severity of the index condition measures at baseline (to assess the extent to which frailty reflects greater severity of the index condition in the trial)
- Baseline assessment of quality of life.

## Comparing frailty indices with/without cognitive deficits

To assess the impact of the inclusions/exclusion of cognitive deficits within the frailty index, we will construct two frailty indices for each trial. The first will include only ‘physical’ deficits (i.e. all deficits described above except those taken from assessments of cognitive function). The second will combine both the physical deficits and the deficits related to cognitive impairment.

## Summarising frailty in trials

We will aim to summarise the prevalence and degree of frailty within each trial, and across trials for each condition.

As the frailty index is intended to be a continuous measure (i.e. expressed the *degree* of frailty, not the presence of absence of frailty) we will aim to summarise the overall distribution. As the trial data are held within a secure environment, and all data exported are subject to strict conditions of non-disclosure, the most appropriate way to summarise this distribution will depend on the prevalence of frailty observed in the trials. We will plan to summarise the distribution of frailty parametrically and export the parameters of the distribution for each trial from the safe havens. We will also plot the expected distribution (based on the parametrisation) against the observed distribution to assess fit.

We will then use the observed distribution of frailty to model the relationship between age, sex, and severity of the underlying index condition and frailty.

While conceptually a frailty index does not place people into frailty ‘categories’, it is common among frailty index studies to assign cut-offs of the frailty index to denote robust, mildly frail, moderately frail, or severely frail people. These categories are used to aid interpretation of the findings and to describe the number of people with a degree of frailty. The number of categories, and the cut-off points assigned, vary from study-to-study. We propose to use the following categorisation, however this may need to be altered to avoid potential disclosure if some categories contain very few participants (i.e. if a trial has <5 ‘severely frail’ people then this data may not be permitted to be exported from the secure environment):

- Robust (no frailty): 0-0.12
- Mild frailty: 0.12-0.24
- Moderate frailty: 0.24-0.36
- Severe frailty: >0.36

These cut points reflect those used in the electronic frailty index (which is routinely used within primary care in England). The combined category of ‘moderate’ and ‘severe’ frailty (i.e. >0.24) also equates to the ‘frail’ category described in many of the early papers validating the frailty index approach.

## Relationship between frailty and serious adverse events and trial attrition

Trials record all adverse events occurring during the trial period. Certain adverse events are characterised as ‘serious adverse events’ (SAEs). SAEs are those meeting one or more of the following criteria:

- Results in death
- Is life threatening
- Results in hospitalisation
- Results in persistent or significant disability/incapacity
- Is a congenital abnormality/birth defect

SAEs are recorded regardless of their relationship (or lack of relationship) with the trial treatment.

Given that frailty represents an increases susceptibility to decompensation and is known to be associated with mortality and hospital admission, we hypothesise that the rate of SAE will be greater in trial participants with a higher frailty index.

We will aim to model the association between frailty and SAE in the trial setting, adjusting for age, sex, and severity of the underlying index condition.

We propose to use a count-based model (e.g. Poisson or negative binomial regression, depending on model fit). We will explore non-linear relationships between frailty and SAEs using fractional polynomials. We will also assess for interactions between frailty and other covariates.

For trial attrition we will identify participants who did not complete the full trial follow-up for any reason. We will assess relationships between baseline frailty index and trial attrition using logistic regression models, adjusted for age and sex.

References

1. Hoogendijk EO, Afilalo J, Ensrud KE, Kowal P, Onder G, Fried LP. Frailty: implications for clinical practice and public health. *The Lancet.* 2019;394(10206):1365-1375.

2. Clegg A, Young J, Iliffe S, Rikkert MO, Rockwood K. Frailty in elderly people. *The lancet.* 2013;381(9868):752-762.

3. Rockwood K, Mitnitski A. Frailty in Relation to the Accumulation of Deficits. *The Journals of Gerontology: Series A.* 2007;62(7):722-727.

4. Searle SD, Mitnitski A, Gahbauer EA, Gill TM, Rockwood K. A standard procedure for creating a frailty index. *BMC Geriatrics.* 2008;8(1):24.

5. Hanlon P, Butterly E, Lewsey J, Siebert S, Mair FS, McAllister DA. Identifying frailty in trials: an analysis of individual participant data from trials of novel pharmacological interventions. *BMC Med.* 2020;18(1):1-12.

6. Hanlon P, Hannigan L, Fischbacker C, et al. Representation of people with comorbidity and multimorbidity in clinical trials of novel drug therapies; an individual-level participant data analysis. *BMC Medicine (in press).* 2019.

7. Klietz M, Greten S, Wegner F, Hoglinger GU. Safety and Tolerability of Pharmacotherapies for Parkinson's Disease in Geriatric Patients. *Drugs & aging.* 2019;36(6):511-530.

8. Clegg A, Bates C, Young J, et al. Development and validation of an electronic frailty index using routine primary care electronic health record data. *Age and ageing.* 2016;45(3):353-360.

# Parameters of frailty index distribution

Based on Generalised Gamma distribution

Physical deficits only:

|  | Mu | Sigma | Q |
| --- | --- | --- | --- |
| AD dementia trial | -1.35732 | 0.317758 | 0.727526 |
| MCI trial 1 | -1.88304 | 0.44764 | 0.916376 |
| MCI trial 2 | -1.79134 | 0.407695 | 1.160961 |

Physical & cognitive deficits

|  | Mu | Sigma | Q |
| --- | --- | --- | --- |
| AD dementia trial | -1.18759 | 0.244326 | 0.592315 |
| MCI trial 1 | -1.93229 | 0.424138 | 0.6207 |
| MCI trial 2 | -1.83536 | 0.383861 | 0.823746 |
